# Multistate Epidemiology of Histoplasmosis, United States, 2011–2014^1^

**DOI:** 10.3201/eid2403.171258

**Published:** 2018-03

**Authors:** Paige A. Armstrong, Brendan R. Jackson, Dirk Haselow, Virgie Fields, Malia Ireland, Connie Austin, Kimberly Signs, Veronica Fialkowski, Reema Patel, Peggy Ellis, Peter C. Iwen, Caitlin Pedati, Suzanne Gibbons-Burgener, Jannifer Anderson, Thomas Dobbs, Sherri Davidson, Mary McIntyre, Kimberly Warren, Joanne Midla, Nhiem Luong, Kaitlin Benedict

**Affiliations:** Centers for Disease Control and Prevention, Atlanta, Georgia, USA (P.A. Armstrong, B.R. Jackson, C. Pedati, K. Benedict);; Arkansas Department of Health, Little Rock, Arkansas, USA (D. Haselow, V. Fields, V. Fialkowski);; Minnesota Department of Health, Saint Paul, Minnesota, USA (M. Ireland);; Illinois Department of Public Health, Springfield, Illinois, USA (C. Austin);; Michigan Department of Health and Human Services, Lansing, Michigan, USA (K. Signs, V. Fialkowski);; Indiana State Department of Health, Indianapolis, Indiana, USA (R. Patel);; Kentucky Department for Public Health, Frankfort, Kentucky, USA (P. Ellis);; University of Nebraska Medical Center, Omaha, Nebraska, USA (P.C. Iwen);; Nebraska Department of Health and Human Services, Lincoln, Nebraska, USA (C. Pedati);; Wisconsin Department of Health Services, Madison, Wisconsin, USA (S. Gibbons-Burgener);; Mississippi State Department of Health, Jackson, Mississippi, USA (J. Anderson, T. Dobbs);; Alabama Department of Public Health, Montgomery, Alabama, USA (S. Davidson, M. McIntyre);; Pennsylvania Department of Health, Harrisburg, Pennsylvania, USA (K. Warren);; Ohio Department of Health, Columbus, Ohio, USA (J. Midla);; Delaware Division of Public Health, Dover, Delaware, USA (N. Luong); 1 This work was presented in part at the 2016 IDWeek Conference, the 66th Epidemic Intelligence Service Conference, and the 2017 Council of State and Territorial Epidemiologists Annual Conference.

**Keywords:** mycoses, fungi, histoplasmosis, epidemiology, surveillance, United States

## Abstract

Histoplasmosis is one of the most common mycoses endemic to the United States, but it was reportable in only 10 states during 2016, when a national case definition was approved. To better characterize the epidemiologic features of histoplasmosis, we analyzed deidentified surveillance data for 2011–2014 from the following 12 states: Alabama, Arkansas, Delaware, Illinois, Indiana, Kentucky, Michigan, Minnesota, Mississippi, Nebraska, Pennsylvania, and Wisconsin. We examined epidemiologic and laboratory features and calculated state-specific annual and county-specific mean annual incidence rates. A total of 3,409 cases were reported. Median patient age was 49 (interquartile range 33–61) years, 2,079 (61%) patients were male, 1,273 (57%) patients were hospitalized, and 76 (7%) patients died. Incidence rates varied markedly between and within states. The high hospitalization rate suggests that histoplasmosis surveillance underestimates the true number of cases. Improved surveillance standardization and surveillance by additional states would provide more comprehensive knowledge of histoplasmosis in the United States.

## Abstract 2

Increased awareness could lead to appropriate diagnosis, prompt treatment, and better patient outcomes.

Histoplasmosis is an infection caused by the soil-dwelling thermally dimorphic fungus *Histoplasma capsulatum* (*1*). Infection typically results from inhalation of aerosolized spores. Only 1% of sporadic infections are estimated to be symptomatic, although attack rates during outbreaks have been as high as 50%–100%, possibly from high-dose exposure (*2*). Most symptomatic infections involve primary pulmonary disease; however, extrapulmonary and severe disseminated disease can occur, especially in immunosuppressed persons.

Histoplasmosis is often described as the most common mycosis endemic to North America (*1*), although data to support this statement are limited, given a lack of national public health surveillance. Although once thought to be endemic to a relatively narrow geographic area, histoplasmosis has been increasingly detected in many parts of the world (*3*,*4*). According to histoplasmin skin test surveys performed in the 1950s and 1960s in the United States, areas surrounding the Ohio and Mississippi River Valleys are recognized as the regions of predominant histoplasmosis endemicity (*5*). However, locally acquired infections have been described outside these areas, suggesting that the geographic range of *Histoplasma* in the United States is wider than is often appreciated (*6*). This incomplete knowledge about geographic areas of risk could deter clinicians from considering histoplasmosis as a cause of illness, leading to misdiagnosis and inappropriate treatment.

A key data source for information about the current geographic distribution and epidemiology of histoplasmosis is disease surveillance. As of 2016, histoplasmosis was reportable in 10 states but not notifiable nationally. Reportable diseases are those that healthcare providers and laboratories are required to report to state, territorial, or local public health authorities. Each jurisdiction’s regulation or law determines which diseases are reportable. For diseases selected as nationally notifiable by the Council of State and Territorial Epidemiologists (CSTE), jurisdictions voluntarily notify the Centers for Disease Control and Prevention (CDC) of cases, and data are summarized for national surveillance (*7*). Until 2016, no national case definition existed for histoplasmosis surveillance. Given this void, each state implemented different case definitions, which generally included similar clinical and laboratory criteria (Technical Appendix).

Current knowledge about this disease and its geographic risk is limited. To improve understanding of the burden of histoplasmosis in the United States, we collected and summarized surveillance data from 12 states.

## Methods

To create a multistate dataset, we combined deidentified data on histoplasmosis cases reported during 2011–2014 from the 10 states where histoplasmosis was reportable in 2016 (Arkansas, Delaware, Illinois, Indiana, Kentucky, Michigan, Minnesota, Nebraska, Pennsylvania, and Wisconsin) and from 2 states where it previously had been reportable (Alabama and Mississippi, which both removed histoplasmosis from their list of reportable diseases in 2015). For states that classified cases as confirmed, probable, or suspected, we included only confirmed and probable cases; from states that did not use such a classification system, we included all cases. Although histoplasmosis is not reportable in Ohio, a comparable convenience sample of data received from reference laboratories was available for 2012–2015 and was analyzed separately.

Because data from each state were collected in different formats, we implemented the following rules to standardize data for analysis. We included variables collected by at least 3 states. Because of inconsistent availability of detailed data, we considered all histoplasmosis laboratory test results recorded as positive to be positive even without an explicitly stated qualitative or quantitative result. Immunodiffusion test results indicating H band, M band, or both were considered positive. Complement fixation titers to the yeast-phase or mycelial-phase antigen >1:8 were considered positive, and, for patients for whom >1 complement fixation titer was available, we retained the highest titer for analysis. We created dichotomous variables to indicate whether positive test results for histoplasmosis were obtained by enzyme immunoassay (of serum, urine, or another or unspecified specimen type), immunodiffusion, complement fixation, PCR, culture, microscopy, or other or unspecified histoplasmosis test. Data for negative histoplasmosis test results were not routinely available and were therefore not included.

We calculated state-specific annual incidence and county-level mean annual incidence per 100,000 persons by using yearly population estimates from the US Census Bureau (https://www.census.gov). County-level incidence estimates represent patients’ county of residence (or, in Ohio, the county of the facility that ordered the laboratory test). To identify factors significantly associated with hospitalization or death, we performed bivariable analyses using χ^2^, Fisher exact, and *t*-tests at p<0.05. We calculated 95% CIs for relative risks.

## Results

### Descriptive Analysis Results

During 2011–2014, a total of 3,409 histoplasmosis cases were reported from 12 states (Table 1). Median patient age was 49 (range 0–94, interquartile range [IQR] 33–61) years, and most (2,079 [61%]) patients were male. Of the 1,729 patients in 8 states that contributed race data, 1,079 (62%) were white, 446 (26%) were of unknown race, and 166 (10%) were black. Of the 1,620 patients in these 8 states for whom ethnicity data were available 1,072 (66%) were non-Hispanic or Latino, 503 (31%) were of unknown ethnicity, and 45 (3%) were Hispanic or Latino. Of the 2,542 patients in 10 states for whom case status was assigned, 1,465 (58%) had confirmed and 1,077 (42%) had probable cases.

**Table 1:** Patient characteristics for 3,409 histoplasmosis cases reported to public health departments, 12 US states, 2011–2014*

Characteristic, no. patients, no. states contributing information	No. (%)
Sex, 3,405 patients, 12 states	
M	2,079 (61.1)
F	1,323 (38.9)
Unknown	3 (0.1)
Race, 1,729 patients, 8 states	
White	1,079 (62.4)
Black	166 (9.6)
Other	24 (1.4)
Asian or Pacific Islander	9 (0.5)
American Indian or Alaska Native	5 (0.3)
Unknown	446 (25.8)
Ethnicity, 1,620 patients, 8 states	
Non-Hispanic or Latino	1,072 (66.2)
Hispanic or Latino	45 (2.8)
Unknown	503 (31.1)
Case status, 2,542 patients, 10 states	
Confirmed	1,465 (57.6)
Probable	1,077 (42.4)
Outbreak-associated illness, 816 patients, 3 states	
Yes	110 (13.5)
No	195 (23.9)
Unknown	511 (62.6)
Immunocompromised, 1,154 patients, 3 states	
Yes	344 (29.8)
No	649 (56.2)
Unknown	161 (14.0)
Hospitalized, 2,218 patients, 9 states	
Yes†	1,273 (57.4)
No	851 (38.4)
Unknown	94 (4.2)
Died, 1,142 patients, 8 states	
Yes	76 (6.7)
No	906 (79.3)
Unknown	160 (14.0)

*Median (range) age for 3,401 patients from 12 states was 49 (0–94) y.  †Median (range) duration for 548 patients from 9 states was 7 (1–126) d.

Symptom data were available from 4 states. The most common symptoms were cough (67% [range by state 56%–81%]), shortness of breath (64% [range 50%–77%]), and fever (56% [range 46%–66%]). Data on immune status were available for 1,154 patients from 3 states; of these, 649 (56%) patients were not immunocompromised, 344 (30%) were immunocompromised, and immune status was unknown for 161 (14%). Hospitalization data were available for 2,218 patients. More than half (1,273 [57%]) of patients were hospitalized; median hospitalization duration for 548 patients for whom hospitalization duration was known was 7 (range 1–126, IQR 4–138) days. Mortality data were available for 1,142 patients; 76 (7%) died.

Three states reported whether cases were associated with an outbreak (816 patients); association for 511 (63%) was unknown, 195 (24%) were not associated, and 110 (14%) were associated (range by state 3%–45%). Exposure data were collected by 3 other states. In Michigan, 29% of patients reported exposure to bird or bat droppings in the 6 weeks before symptom onset; in Illinois, 24% of patients had exposure to “large quantities of bird/bat droppings”; and in Pennsylvania, 8% of interviewed patients noted “contact with bird/bat droppings.”

Nine states contributed laboratory data (Table 2). Of 1,929 patients with any positive histoplasmosis test result, antigen test results were positive for 644 (33%), antibody test results were positive for 1,052 (55%), and culture results were positive for 257 (13%). Of the 644 patients with a positive antigen test result, 536 (83%) had tests performed on urine specimens, 146 (23%) had tests performed on serum, and 42 (7%) had tests performed on a specimen of unspecified type. Of the 1,052 patients with a positive antibody test result, antibodies were detected in 618 (59%) patients by an immunodiffusion test and in 849 (81%) by a complement fixation test. The median highest complement fixation titer was 1:64 (range 1:8–1:4,096). Positive results for other positive histoplasmosis tests not mentioned above or that could not be classified as a specific test type were reported for 248 (13%) patients.

**Table 2:** Positive histoplasmosis laboratory test results among 1,929 histoplasmosis cases reported to public health departments, 9 US states, 2011–2014*

Test type	No. (%)
Antigen	644 (33.4)
Urine	536 (27.8)
Serum	146 (7.6)
Unspecified specimen type	248 (12.9)
Antibody	1,052 (54.5)
Immunodiffusion	618 (32.0)
Complement fixation	849 (44.0)
Confirmatory and histopathology	262 (13.5)
PCR	5 (0.3)
Culture	257 (13.3)
Microscopy	24 (1.3)
Other or unspecified type	248 (12.9)

*For some patients, >1 type of test was performed.

Ohio contributed data on 303 histoplasmosis cases. Median patient age was 53 (range 6–92, IQR 40–67) years. Most (183 [61%]) patients were male. Positive antigen test results were reported for 128 (42%) patients (87 urine and 41 unspecified specimen type), positive antibody test results for 129 (43%) (127 complement fixation and 2 immunodiffusion), and positive other or unspecified test types for 46 (15%).

### Bivariable Analysis Results

Factors significantly associated with hospitalization were age >50 years (relative risk 1.23, 95% CI 1.14–1.32); male sex (1.08, 95% CI 1.01–1.45); nonwhite race (1.26, 95% CI 1.13–1.41); immunocompromised status (1.78, 95% CI 1.62–1.96); and positive antigen test result (1.75, 95% CI 1.62–1.89) or confirmatory test result (1.21, 95% CI 1.09–1.34) (Table 3). Patients with a positive antibody test result were less likely to be hospitalized than those without a positive antibody test result (0.58, 95% CI 0.53–0.63). Factors significantly associated with death were age >50 years (6.28, 95% CI 3.43–11.49), immunocompromised status (6.07, 95% CI 2.61–14.11), positive antigen test result (1.73, 95% CI 1.04–2.87), or positive confirmatory test result (2.13, 95% CI 1.28–3.54). Patients with a positive antibody test result were less likely to die than those without a positive antibody test result (0.41, 95% CI 0.24–0.71).

**Table 3:** Patient factors associated with hospitalization or death among histoplasmosis cases reported to public health, 12 US states, 2011–2014*

Characteristic	Hospitalization			Death	
	RR (95% CI)	p value		RR (95% CI)	p value
Age >50 y	1.23 (1.14–1.32)	<0.001		6.28 (3.43–11.49)	<0.001
Male sex	1.08 (1.01–1.45)	0.033		1.02 (0.65–1.58)	0.944
Nonwhite race†	1.26 (1.13–1.41)	<0.001		0.82 (0.36–1.86)	0.627
Immunocompromised‡	1.78 (1.62–1.96)	<0.001		6.07 (2.61–14.11)	<0.001
Positive laboratory test result§					
Antigen	1.75 (1.62–1.89)	0.001		1.73 (1.04–2.87)	0.033
Antibody	0.58 (0.53–0.63)	<0.001		0.41 (0.24–0.71)	0.001
Confirmatory	1.21 (1.09–1.34)	0.001		2.13 (1.28–3.54)	0.003

*RR, relative risk. †Data collected by 8 states. ‡Data collected data by 3 states. §Data collected by 9 states.

### Incidence

**Figure 1:**
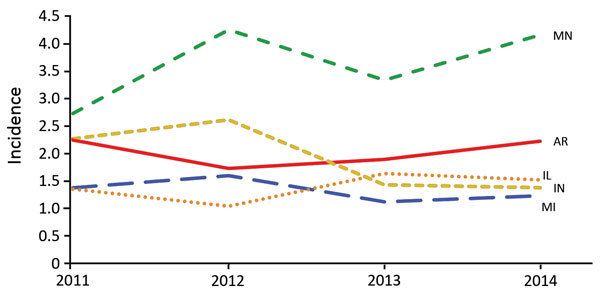
Annual state-specific histoplasmosis incidence (no. cases/100,000 population) for the 5 US states in which incidence was highest, 2011–2014.

**Figure 2:**
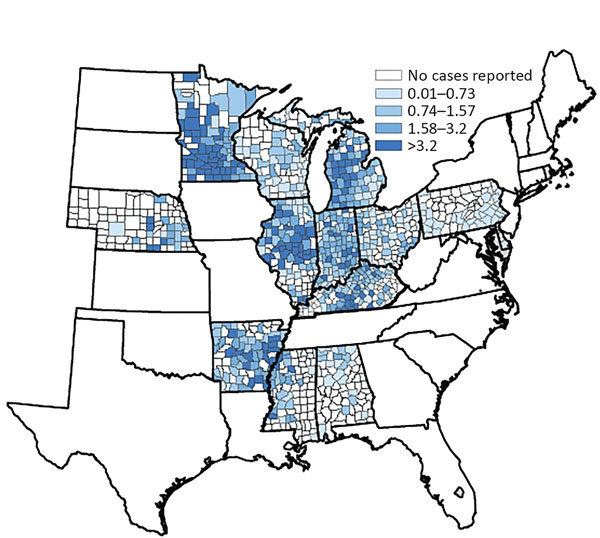
County-specific histoplasmosis incidence (no. cases/100,000 population) for the 12 US states from which surveillance data were available, 2011–2014.

Annual incidence rates were highest for Arkansas, Illinois, Indiana, Michigan, and Minnesota (Figure 1). State-specific annual incidence rates ranged from 0 to 4.3 cases/100,000 population, and no consistent increases or decreases occurred over the 4-year period. Mean county-level incidence ranged from 0 to 39 cases/100,000 population by county (Figure 2).

## Discussion

This summary of 2011–2014 state-based public health surveillance data on 3,409 histoplasmosis patients in 12 US states provides a broad, population-level epidemiologic description of this underrecognized disease. Key findings include granular data about geographic distribution of the disease, patient demographic features, and common methods of laboratory diagnosis. These data suggest substantial underdetection of histoplasmosis and a need for more standardized histoplasmosis surveillance. By characterizing populations at greater risk, these data can help increase public awareness and help healthcare providers better target diagnostics and early antifungal treatment.

Given that most infected persons are asymptomatic, the high proportions of patients who were hospitalized (57%) and died (7%) suggest relatively severe illness for these patients. In comparison, a review of 105 US histoplasmosis outbreaks during 1938–2013 found that 25% of all outbreak-associated patients were hospitalized and 1% died; hospitalization and death have become less common in recent years (*8*). National hospitalization data, which indicate that >5,000 histoplasmosis admissions (including multiple admissions per patient) occurred during 2012 (*9*), also suggest underdetection and underreporting in our surveillance data. We saw an annual mean of 852 cases from 12 states. Extrapolating from the hospitalization rate of 57% of patients for whom data were available, an estimated ≈485 patients (852 × 0.57) with reported disease would be hospitalized annually. To suggest that all remaining >4,500 estimated national hospitalizations occur in states without surveillance seems unlikely. Rather, the more likely explanation is that substantial underreporting occurs in states in which histoplasmosis is reportable, even among hospitalized patients. Furthermore, a retrospective cohort study in a tertiary care center found that the 6-month all-cause mortality rate among symptomatic histoplasmosis patients was 4% and was associated with older patient age (*10*). Our analysis, which included inpatients and outpatients, showed a higher mortality rate than the 5% in-hospital mortality rate found by an analysis of nationwide histoplasmosis-associated hospitalizations during 2001–2012 (*9*), further supporting the conclusion that current histoplasmosis surveillance captures only a small subset of more severely ill persons. To provide more information about the clinical spectrum, burden, and outcomes of each of the different forms of histoplasmosis, future surveillance could be improved by more consistent collection of data on severity and disease forms (e.g., acute pulmonary, chronic pulmonary, disseminated).

Current maps showing the presumed geographic distribution of histoplasmosis are still primarily informed by large-scale skin testing performed in the 1950s and 1960s, which identified the Ohio and Mississippi River Valleys as having the highest proportion of positive skin test reactions (*5*). In our analysis, we included much of the traditionally defined population at greatest risk; we did not include 2 (Missouri and Tennessee) of the 5 states with areas for which skin test positivity rates were >85%. Again, considering the apparent reporting bias of our data toward more severe cases, underdiagnosis and underreporting were likely. These results also support existing evidence that cases occur outside of the traditionally defined regions (*6*,*11*). Knowledge of histoplasmosis-endemic regions is particularly helpful for clinicians when risk-stratifying their patients and deciding whether to test for this disease. Because of the variability in state-specific case definitions, incidence rates might not be directly comparable between states but are useful for identifying trends within states. For example, Minnesota (only a small portion of which is traditionally considered histoplasmosis endemic) reported an incidence rate nearly double that of the 4 states with the next highest rates, and that rate has remained consistently high over the same period, possibly as a result of a broader case definition and strong surveillance system. Despite these limitations in interstate comparability, data from Minnesota, Wisconsin, and Michigan suggest that histoplasmosis routinely occurs in areas where histoplasmosis was not previously considered endemic. Although interstate travel could account for some cases, higher incidence in the central and northern areas of these states, farther from known disease-endemic areas, suggests that local acquisition is likely. County-level incidence rates are also useful for demonstrating the distribution of cases within states. For instance, in Illinois, incidence was highest in counties clustered in the central region; in Arkansas and Mississippi, incidence was higher in counties along the Mississippi River.

These surveillance data provide a valuable window into the descriptive epidemiology of histoplasmosis in the United States. Although the large proportion of patients for whom race data were missing precluded a comparison of incidence by race, nonwhite patients were more likely than white patients to have been hospitalized, a finding that warrants future study. Black race has been associated with more severe histoplasmosis in patients with AIDS (*12*), but in general, no racial disposition has been documented for histoplasmosis (*3*,*13*) as it has been for coccidioidomycosis. In contrast, a sex disparity was readily evident. Male patients accounted for nearly two thirds of cases and were more likely than female patients to be hospitalized. A similar male predominance was seen in other studies of histoplasmosis and resulting hospitalizations (*3*,*8*–*11*) and for patients with other fungal infections, including coccidioidomycosis and blastomycosis (*11*,*14*,*15*). The reasons for this disparity are not entirely known, although different outdoor recreational and occupational exposures have been suggested (*3*,*14*,*15*). Of note, we did not observe an increased risk for death among male patients. Other studies of death from histoplasmosis in the United States also have not found male sex to be a risk factor for death (*10*,*16*).

In the 3 states with available data, nearly one third of histoplasmosis patients were reported to have been immunocompromised, providing additional evidence that current histoplasmosis surveillance, and perhaps clinical diagnosis, tends to detect more severe cases. We were unable to parse information about specific immunocompromising conditions from surveillance data. However, according to a study of histoplasmosis-associated hospitalizations, HIV infection was the most common concurrent immunocompromising condition listed on histoplasmosis-associated discharge records in the early 2000s; by 2012, diabetes mellitus (21%) had eclipsed HIV infection (17%) (*9*). The proportion of hospitalizations for immune-mediated inflammatory disease (rheumatoid arthritis, inflammatory bowel disease, and psoriasis) listed on discharge records also increased from 4% in 2001 to 10% in 2012, as did the proportion with solid organ or stem cell transplant (from 1% to 6%) (*9*). Standardized surveillance data would provide additional insight into the populations at highest risk for histoplasmosis and could help identify possible prevention opportunities.

Although state case definitions used different laboratory criteria for case classification, the laboratory data reported by 9 states provide a window into the most commonly positive test types and associations with disease outcomes. The fact that culture results were positive for 13% of patients for whom data were available again underscores the bias of detection and reporting toward severe cases because cultures are more often positive for patients with disseminated or chronic pulmonary disease than for those with milder disease (*17*). Accordingly, a positive confirmatory test result was associated with higher risk for hospitalization and death. Of note, a positive antigen test result, reported for one third of patients, was even more strongly associated with hospitalization than was a positive confirmatory test result, although the associations were similar for death. Antigen testing is particularly useful for immunocompromised patients and patients with severe disease, who might not mount an immune response, and is less sensitive for patients with subacute pulmonary disease (*18*). More than half of patients had a positive antibody test result, and these patients were less likely to have been hospitalized or die than those without a positive result, probably because these tests are more sensitive than others for patients with milder disease and might be used more routinely in outpatient settings. Serologic cross-reactions, particularly with antigen testing, and misclassification of blastomycosis cases as histoplasmosis might have occurred in areas in which both diseases are endemic; however, because extensive follow-up to differentiate between the 2 diseases occurred in some areas, the overall contribution of such false-positive histoplasmosis cases in our analysis is probably small.

As with other mycoses endemic to certain areas, assessing risk associated with recreational and occupational exposures is useful for identifying clusters of cases and developing recommendations for subsequent prevention of additional cluster-associated cases. A review of US histoplasmosis outbreaks described the presence of either birds or bats for 77% of outbreaks (*8*). However, limited published data are available about the proportion of sporadic (nonoutbreak) cases resulting from these types of exposures. For our analysis, only 3 states had collected similar data; reports of exposure to birds, bats, and their droppings were reported much less frequently (<30%) for sporadic cases than for outbreaks. Overall, exposure data were assessed for a relatively small portion of cases, and given the differences in how these data were collected, these numbers might not be directly comparable. The frequency of these exposures among the general population is also not known. Although histoplasmosis surveillance might not be able to detect broad exposures that lead to prevention messaging, tracking cases can enable detection of spatial and temporal hotspots and clusters that can lead to more intensive exposure investigations.

Our analysis has limitations inherent in summarizing disparate public health surveillance data. Primarily, differing state case definitions limit most direct comparisons between states. In addition, states collected different data, so denominators differed for many epidemiologic characteristics. Another limitation is the large proportion of missing and unknown data (either because surveillance investigators did not intend to collect specific data or because they did attempt to collect the data but were unable to do so). For this reason, we were not able to perform multivariable analyses. We were also not able to determine whether deaths were associated with histoplasmosis. Ultimately, more standardized histoplasmosis surveillance data would enable a better understanding of this disease by facilitating comparisons across states. The standardized surveillance case definition approved by CSTE in June 2016 will probably enable more consistent comparisons of incidence and trends in states that use this case definition (*19*).

This multistate comparison of histoplasmosis surveillance data is a first step toward an updated understanding of the burden of this disease in the United States. Other actions that would improve our understanding of histoplasmosis include expanding the number of states in which it is reportable and making it nationally notifiable. Although underreporting of milder cases might explain the high severity of disease among reported cases, the fact that laboratory-based reporting is common suggests that at least some of the skewed disease spectrum results from underdiagnosis. Increased awareness about histoplasmosis among the public, the public health community, and healthcare providers could improve diagnosis, leading to appropriate treatment and better patient outcomes and reducing harm from administering multiple courses of antibacterial drugs ineffective against fungi, as commonly occurs for coccidioidomycosis (*20*,*21*). To identify populations at highest risk and opportunities for prevention, additional study of the incidence and epidemiologic, clinical, and laboratory features of histoplasmosis cases nationwide is needed.

Technical Appendix Laboratory criteria used by states for confirmation of histoplasmosis; state case definitions for histoplasmosis, United States, 2011–2014.
