# Supplementary material for: Multistate Epidemiology of Histoplasmosis, United States, 2011–20141
Source: Emerg Infect Dis. 2018 Mar;24(3):425–31. doi: 10.3201/eid2403.171258 (PMC5823339; doi:10.3201/eid2403.171258)
Supplement: Technical Appendix — Laboratory criteria used by states for confirmation of histoplasmosis; state case definitions for histoplasmosis, United States, 2011–2014. [file 17-1258-Techapp-s1.pdf]

Article DOI: https://doi.org/10.3201/eid2403.171258

Multistate Histoplasmosis Surveillance, United States, 2011–2014

Technical Appendix

Technical Appendix Table. Laboratory criteria used by states for confirmation of histoplasmosis, 2011–2014*

Laboratory criteria	State									
	AR	DE	IL	IN	KY	MI	MN	NE	PA	WI
Culture	X	X	X	X	X	X	X	X	X	X
Histopathology	X	X	X	X	X	X	X	X	X	X
Rise in CF titer	X	X	X	X	X	X	X	X	X	X
H-band	X		X	X						
M-band seroconvert			X				X	X		
Nucleic acid			X					X		
Cytopathology										
Single CF titer		X	X	X			X	X		
M-bands			X				X	X		
Antigen	X	X	X	X	X		X	X		

*A checkmark indicates that laboratory criteria was used as case confirmation for histoplasmosis during 2011–2014.
†Based on laboratory criteria as defined by the 2016 CSTE case definition for histoplasmosis.
‡Any positive serologic test considered confirmatory.
§These laboratory criteria are defined as non-confirmatory by the 2016 CSTE case definition.

State case definitions for histoplasmosis, 2011–2014

Arkansas

A. CLINICAL DESCRIPTION: A systemic fungal infection of varying severity caused by *Histoplasma capsulatum*. Infection may be asymptomatic or take one of four clinical forms:

• Acute benign respiratory - mild respiratory illness with general malaise, fever, chills, headache, myalgia, chest pains, nonproductive cough and scattered small calcifications of the lung.

• Acute disseminated - debilitating fever, GI symptoms, bone marrow suppression, lymphadenopathy. Most frequent in children and immunosuppressed; fatal if not treated.

• Chronic pulmonary - clinically and radiologically resembles chronic pulmonary tuberculosis with cavitations, usually in middle-aged and elderly persons with underlying emphysema.

• Chronic disseminated - low-grade fever, weight loss, weakness, liver and spleen enlargement, mucosal ulcers, subacute course with slow progression; fatal if not treated.

B. REPORTING CRITERIA: Laboratory confirmation.

C. LABORATORY CRITERIA FOR CONFIRMATION:

• Isolation of H. capsulatum from culture of bone marrow, sputum, or lesions, OR

• Histologic demonstration of intracellular yeast cells from bone marrow or tissue biopsy, OR

• Detection of H. capsulatum polysaccharide antigen in urine or serum, OR

• Rise in CF titers to either histoplasmin or yeast-phase antigen.

• Positive serology test for anti-H band antibody

Delaware

Clinical features:

A systemic mycosis of varying severity, with the primary lesion usually in the lungs. Clinical manifestation ranges from asymptomatic to minor self-limited or life-threatening illnesses. Four clinical forms are recognized:

• Acute respiratory: Varies from a mild respiratory illness to temporary incapacity with general malaise, fever, chills, headache, myalgia, chest pains and nonproductive cough; occasional erythema multiforme and erythema nodosum. Multiple, small scattered calcifications in the lung, hilar lymph nodes, spleen and liver may be late findings.

• Acute disseminated: with debilitating fever, GI symptoms, evidence of bone marrow suppression, hepatosplenomegaly, and lymphadenopathy. A rapid course, most frequent in infants and young children and immunocompromised patients. Without treatment, usually fatal.

• Chronic disseminated: with low-grade intermittent fever, weight loss, weakness, hepatosplenomegaly, mild hematological abnormalities and focal manifestations of disease. Subacute course progressing over 10–11 months. Usually fatal unless treated.

• Chronic pulmonary form: Clinically and radiologically resembles chronic pulmonary tuberculosis with cavitation. Most often in middle-aged and elderly men with underlying

emphysema, progresses over months or years, with periods of quiescence and sometimes spontaneous cure

Laboratory criteria for confirmation: Clinical diagnosis is confirmed by

• Culture, DNA probe, or by visualizing the fungus in Giemsa- or Wright-stained smears of the ulcer exudates, bone marrow, blood, sputum.

• Demonstration of the fungus in biopsies of ulcers, liver, lymph nodes, lung or bone marrow

• Serologic test-immunodiffusion test: A rise in complement fixation titers in paired sera may occur early in acute infection and is suggestive evidence of active disease. A titers of 1:32 or greater is suggestive of active disease

• Detection of antigen in serum or urine: useful in making the diagnosis and following the results of treatment for disseminated histoplasmosis

Case definition: A case is confirmed if clinically compatible with laboratory test result(s)

Illinois

Clinical Case Definition

A case of acute respiratory histoplasmosis is defined as an influenza-like illness with two or more of the following symptoms: fever, cough, shortness of breath or chest pain. Acute histoplasmosis may occur in the absence of respiratory symptoms. The symptoms of acute disseminated histoplasmosis include fever and weight loss.

Laboratory Criteria for Diagnosis

1. Isolation of *H. capsulatum* from a clinical specimen by culture

2. 4-fold rise in complement fixation titers (yeast or mycelial) collected 2–4 weeks apart

3. M or H precipitin bands positive by immunodiffusion

4. A single complement fixation titer (year or mycelial) of > = 1:32 (e.g., 1:32, 1:64, 1:128, etc. Note: 1:8, 1:6 are <1:32)

5. Demonstration of *H. capsulatum* polysaccharide antigen by radioimmunoassay or enzyme immunoassay in blood, urine or other body fluid

6. Demonstration of *H. capsulatum* antibody by enzyme immunoassay in serum

7. Demonstration of *H. capsulatum* (by histopathology or silver staining) in blood, biopsy material, or other body fluid

8. Detection of *H. capsulatum* by DNA probe on a respiratory specimen

Note: “*Histoplasma* Galactomannan Antigen Quantitative by EIA” is a type of test that detects the galactomannan (a polysaccharide) from *H. capsulatum*. Galactomannan is the specific antigen in this test; it is not a different species of *Histoplasma*.

Case Classification

Acute Respiratory Histoplasmosis

• Probable: A case that is clinically compatible, not culture confirmed, does not have a 4-fold rise in titer, but positive by any of the other laboratory methods listed above (numbers 3–8 in laboratory criteria). A case is also considered probable with clinically compatible symptoms, no laboratory confirmation, and an epi link to a suspected source during an outbreak.

• Confirmed: A patient that is clinically compatible, and culture positive for *H. capsulatum* or has a 4-fold rise in titers collected 2–4 weeks apart (numbers 1 or 2 in laboratory criteria).

Acute Disseminated Histoplasmosis

• Probable: A case that has fever and weight loss with or without respiratory symptoms, and evidence of *H. capsulatum* by either histopathology staining or DNA probe of a specimen from an extrapulmonary site*.

• Confirmed: A case that has fever and weight loss with or without respiratory symptoms, and a positive culture from an extrapulmonary site*.

* including blood

Indiana

Clinical disease

• Asymptomatic - individual has no clinical signs or symptoms, but has immunological evidence of infection. This is a common clinical disease presentation in Indiana and is not reportable as histoplasmosis.

• Acute disseminated - is an illness of short duration that involves other organs in addition to the lungs. It is marked by cough, exhaustion and enlargement of the liver and spleen.

• Acute benign respiratory - characterized by weakness, fever, chest pains, and cough. Symptom severity is dependent on magnitude of exposure to fungal conidia.

• Chronic disseminated - a prolonged illness involving organs other than the lungs. It may include by fever, anemia, hepatitis, pneumonia, endocarditis, meningitis, and ulcers of the mouth, tongue, nose, and larynx.

• Chronic pulmonary - resembles tuberculosis

Laboratory test used in confirming case

• Culture - a positive culture for *Histoplasma capsulatum* is sufficient for case confirmation.

• Histological - any pathological finding indicating an infection with *Histoplasma* is sufficient for case confirmation

• Complement-fixing antibodies - presence of antibodies to yeast (Y) or mycelial (M) antigens in dilutions greater than I: 16 in patients with a compatible clinical presentation and no other explanation for his/her illness is sufficient for case confirmation

• Immunodiffusion testing - The presence of H band antibodies is indicative of a recent infection (within 6 mo.). The presence of M band antibodies indicates a histoplasmosis infection and they may persist for years. The presence of H band antibodies (with or without M band) with a compatible clinical presentation and no other explanation for his/her illness is sufficient for case confirmation. H antibodies will become undetectable after 6 months with uncomplicated disease. The presence of M band antibodies without H band antibodies or compatible clinical presentation represents an old infection and is not reportable.

• Serum or urine antigen - A positive test with a compatible clinical presentation is sufficient for case confirmation.

Kentucky

CLINICAL DESCRIPTION:

A systemic fungal infection of varying severity caused by *Histoplasma capsulatum*. Infection may be asymptomatic or take one of four clinical forms:

• Acute benign respiratory - mild respiratory illness with general malaise, fever, chills, headache, myalgia, chest pains, nonproductive cough and scattered small calcifications of the lung.

• Acute disseminated - debilitating fever, GI symptoms, bone marrow suppression, lymphadenopathy. Most frequent in children and immunosuppressed; fatal if not treated.

• Chronic pulmonary - clinically and radiologically resembles chronic pulmonary tuberculosis with cavitations, usually in middle-aged and elderly persons with underlying emphysema

• Chronic disseminated - low-grade fever, weight loss, weakness, liver and spleen enlargement, mucosal ulcers, subacute course with slow progression; fatal if not treated.

LABORATORY CRITERIA FOR CONFIRMATION:

• Isolation of *H. capsulatum* from culture of bone marrow, sputum, or lesions, OR

• Histological demonstration of intracellular yeast cells from bone marrow or tissue biopsy, OR

• Detection of *H. capsulatum* polysaccharide antigen in urine or serum, OR

• Rise in CF titers to either histoplasmin or yeast-phase antigen.

COMMENT:

Positive histoplasmin skin test IS NOT sufficient evidence.

REPORTING CRITERIA:

Signs/symptoms and/or laboratory confirmation

Michigan

In order for a patient to be considered to have an acute case of histoplasmosis, they must have a clinically compatible illness coupled with laboratory evidence of infection. Please use the following case definition when classifying cases for MDSS entry:

Clinical Description

A case of acute histoplasmosis is defined as an influenza-like illness with two or more of the following symptoms: fever/chills, cough, chest pain, weakness, or myalgia/arthralgia.

Laboratory Criteria for Diagnosis

Probable:

- Complement fixation titer to the yeast-phase antigen ≥1:32 or
- H band detected by Immunodiffusion testing or
- Detection of antigen in body fluids including urine, serum, cerebral spinal fluid, and broncho-alveolar lavage fluid

Confirmed:

- A 4-fold rise in compliment fixation titer between serum specimens collected 2–4 weeks apart
- Identification of the organism in tissues by histopathology
- Isolation of the organism from cultures

Minnesota

The case definition is based on positive laboratory results in patients with clinical evidence of disease.

A confirmed case is defined as a Minnesota Resident with at least one of the following:

- positive Immune diffusion (ID) test with an H band,
- positive ID test with an M band,
- 4-fold or greater rise in titer on Complement fixation (CF),
- single CF titer of ≥1:32 (can be yeast Ab and/or mycelial Ab),

• positive culture,

• positive serum or urine antigen test >0.4 ng/mL, or

• positive histochemical identification of clinical pathology specimens ex: bronchial lavage

A case is invalid if:

• the individual is not a Minnesota Resident,

• single Complement fixation titer is <1:32, or

• test result was a fungus or disease other than Histoplasmosis

In outbreak cases involving investigation, in addition to a positive laboratory result, cases must have one or more of the following clinical signs: shortness of breath, weakness, fever, cough, decreased appetite, chest pain, weight loss, night sweats, headache, difficulty swallowing, enlarged lymph nodes, hemoptysis or seizures.

Nebraska

Confirmed Case Definition – Acute

Illness as described below:

• If general symptoms are apparent, they may include:

o General ill feeling

o Fever

o Chest pains

o Dry or nonproductive cough

• Forms of the disease:

o Acute Benign

▪ Flu-like symptoms

▪ Tiredness

▪ Fever

▪ Chills

▪ Headache

▪ Muscle aches

▪ Chest pains

▪ Non-productive cough

o Acute Disseminated

▪ Fever

▪ Vomiting or diarrhea

▪ Enlarged lymph nodes and/or spleen

▪ May be fatal without treatment

▪ Usually occurs in infants, young children and the immunocompromised

o Chronic disseminated

▪ Fever

▪ Weight loss

▪ Weakness

▪ Enlarged liver and spleen

▪ Mild blood abnormalities

▪ Heart and/or meninges may also be affected

▪ May develop ulcers of the mouth, larynx, stomach, or bowel

▪ Usually develops over 10–11 months

▪ Usually fatal if left untreated

o Chronic pulmonary

▪ Resembles TB (symptomatically and on chest x-ray)

▪ Night sweats

▪ Weight loss

▪ Loss of appetite

▪ Chronic cough (lasting longer than 3 weeks)

▪ Occurs most often in middle-aged and elderly men with other lung diseases

• One positive laboratory test

o Serology

o Urine antigen

o Histopathology

o Culture

Presumptive Case Definition

• Epidemiologic link to a confirmed case, even without laboratory confirmation

Pennsylvania

Clinical criteria

• Acute illness with two or more of the following: fever/chills, cough, chest pain, weakness, arthralgias/myalgias; **OR**

• Physician diagnosis of Histoplasmosis

Laboratory criteria

• Confirmatory

o 4-fold rise in titer by complement fixation (CF) in 2 serum specimens taken 2–4 weeks apart; **OR**

o Identification of *H. capsulatum* in tissue by histopathology; **OR**

o Isolation of *H. capsulatum* from culture

• Supportive

• CF titer of >1:32 in a single serum specimen; **OR**

• H band detection by immunodiffusion testing; **OR**

• Antigen detection in a clinical specimen

Case Classification Categories

Confirmed: A case that meets the clinical case definition and is laboratory confirmed

Probable: A case that meets the clinical case definition and has only supportive laboratory evidence of infection

Wisconsin

CLINICAL DESCRIPTION: A systemic fungal infection of varying severity caused by

Histoplasma capsulatum. Infection may be asymptomatic or take one of four clinical forms:

• Acute benign respiratory - mild respiratory illness with general malaise, fever, chills, headache, myalgia, chest pains, nonproductive cough and scattered small calcifications of the lung.

• Acute disseminated - debilitating fever, GI symptoms, bone marrow suppression, lymphadenopathy. Most frequent in children and immunosuppressed; fatal if not treated.

• Chronic pulmonary - clinically and radiologically resembles chronic pulmonary tuberculosis with cavitations, usually in middle-aged and elderly persons with underlying emphysema.

• Chronic disseminated - low-grade fever, weight loss, weakness, liver and spleen enlargement, mucosal ulcers, subacute course with slow progression; fatal if not treated.

LABORATORY CRITERIA FOR CONFIRMATION

Confirmatory

• Isolation of *H. capsulatum* from culture of bone marrow, sputum, or lesions

• Histologic demonstration of intracellular yeast cells from bone marrow or tissue biopsy

• Rise in CF titers to either histoplasmin or yeast-phase antigen.

Supportive

• Positive serology test for anti-H band antibody

• Detection of *H. capsulatum* polysaccharide antigen in urine or serum, AND is epidemiologically linked to a confirmed histoplasmosis outbreak or cluster.

D. WISCONSIN CASE DEFINITION

Confirmed: Clinically compatible illness with confirmatory laboratory evidence.

Probable: Clinically compatible illness with supportive laboratory evidence.
